# Determining the longitudinal accuracy and reproducibility of T_1_ and T_2_ in a 3T MRI scanner

**DOI:** 10.1002/acm2.13432

**Published:** 2021-09-25

**Authors:** Madeline E. Carr, Kathryn E. Keenan, Robba Rai, Peter Metcalfe, Amy Walker, Lois Holloway

**Affiliations:** ^1^ Centre for Medical and Radiation Physics University of Wollongong Wollongong Australia; ^2^ Ingham Institute for Applied Medical Research Liverpool Australia; ^3^ Liverpool and Macarthur Cancer Therapy Centres Sydney Australia; ^4^ National Institute of Standards and Technology Boulder Colorado USA; ^5^ South Western Sydney Clinical School University of New South Wales Liverpool Australia; ^6^ Institute of Medical Physics University of Sydney Camperdown Australia

**Keywords:** accuracy, NIST, phantom, qMRI, relaxation, reproducibility

## Abstract

**Purpose:**

To determine baseline accuracy and reproducibility of T_1_ and T_2_ relaxation times over 12 months on a dedicated radiotherapy MRI scanner.

**Methods:**

An International Society of Magnetic Resonance in Medicine/National Institute of Standards and Technology (ISMRM/NIST) System Phantom was scanned monthly on a 3T MRI scanner for 1 year. T_1_ was measured using inversion recovery (T_1_‐IR) and variable flip angle (T_1_‐VFA) sequences and T_2_ was measured using a multi‐echo spin echo (T_2_‐SE) sequence. For each vial in the phantom, accuracy errors (%bias) were determined by the relative differences in measured T_1_ and T_2_ times compared to reference values. Reproducibility was measured by the coefficient of variation (CV) of T_1_ and T_2_ measurements across monthly scans. Accuracy and reproducibility were mainly assessed on vials with relaxation times expected to be in physiological ranges at 3T.

**Results:**

A strong linear correlation between measured and reference relaxation times was found for all sequences tested (*R* ^2 ^> 0.997). Baseline bias (and CV[%]) for T_1_‐IR, T_1_‐VFA and T_2_‐SE sequences were +2.0% (2.1), +6.5% (4.2), and +8.5% (1.9), respectively.

**Conclusions:**

The accuracy and reproducibility of T_1_ and T_2_ on the scanner were considered sufficient for the sequences tested. No longitudinal trends of variation were deduced, suggesting less frequent measurements are required following the establishment of baselines.

## 1 INTRODUCTION

Quantitative magnetic resonance imaging (qMRI) utilizes MR methods that allow for measurements of physiological changes in physical units. Longitudinal (T_1_) and transversal (T_2_) relaxation times are examples of physical properties able to be measured using qMRI. The T_1_ of a tissue is generally measured in parallel with dynamic contrast‐enhanced MRI sequences to quantify blood perfusion,^1^, ^2^, ^3^ and T_2_ has been used in applications such as disease diagnosis^3^, ^4^ and detecting cartilage degeneration.^5^ Quantifying the changes in T_1_ and T_2_ over time has seen the potential to monitor treatment responses.^3^, ^4^ For qMRI to have wide‐spread clinical applications, it is essential that the parameters being derived are accurate, repeatable, reproducible and independent of scanner performance.^6^, ^7^ Phantom‐based quality assurance (QA) programs can assist in determining the qMRI methods’ technical performance on a specific scanner.

The International Society of Magnetic Resonance in Medicine/National Institute of Standards and Technology (ISMRM/NIST) System Phantom is commercially available for the execution of qMRI QA programs. It can measure both clinical scanner properties (e.g., SNR and geometric distortions) and a wide range of human relevant T_1_ and T_2_ relaxation times.^8^, ^9^, ^10^, ^11^ In past longitudinal studies, this phantom was scanned repeatedly (up to 100 days) to monitor changes in T_1_ and T_2_ over time using Magnetic Resonance Fingerprinting.^12^, ^13^ Variability in T_1_ derived from traditional spin echo (SE) methods has also been assessed using the phantom: single center results found that accuracy and reproducibility of T_1_ varied pre‐ and post‐scanner upgrade.^14^ Further, a multi‐site study found these properties to be dependent on sample T_1_ relaxation time, magnetic field strength, and sequence choice.^1^

Previous longitudinal studies assessing T_1_ and T_2_ accuracy and reproducibility acquired measurements at infrequent and/or over short time periods,^1^, ^14^, ^15^ or assessed relaxation times relevant to specific anatomy at different magnetic field strengths.^16^ This study aimed to deduce scanner baseline accuracy and reproducibility for a wide range of T_1_ and T_2_ times by longitudinally monitoring the parameters on a 3T MRI scanner. This was completed by imaging the System Phantom over the course of 1 year at monthly intervals using standardized sequences. Quantifying the changes in parameters such as T_1_ and T_2_ over time is essential for advancing the use of qMRI clinically (e.g., in treatment response monitoring).^7^

## 2 METHODS

### 2.1 Data acquisition

An ISMRM/NIST System Phantom (serial#: 130‐0111: CaliberMRI, Colorado, United States) was imaged monthly for 1 year (at least 2 weeks apart) using a 3T MRI scanner (Siemens Healthineers, MAGNETOM Skyra, Erlangen, Germany). Imaging was completed using a 20 channel Head/Neck coil. T_1_‐weighted inversion recovery (T_1_‐IR) and variable flip angle (T_1_‐VFA) sequences were utilized for T_1_‐mapping, while a multi‐echo spin echo (T_2_‐SE) sequence was used for T_2_‐mapping. Sequence and parameter selections (outlined in Table 1) were based on recommended protocols in the phantom manual.^17^ All phantom setups, image acquisitions, and analyses were completed by one user (physicist with 3 years of MRI experience).

**TABLE 1:** Acquisition parameters utilized for the three sequences tested. This includes T_1_‐inversion recovery (T_1_‐IR), T_1_‐variable flip angle (T_1_‐VFA) and multi‐echo spin echo (T_2_‐SE). Note FA = flip angle, TE = echo time, TR = repetition time, TI = time of inversion, FOV = field of view, FE/PE/SE = frequency/phase/slice encoding, respectively

Parameter	T_1_‐IR	T_1_‐VFA	T_2_‐SE
Sequence	2D/TSE‐IR	3D/SGRE	2D/SE‐ME
Orientation	Coronal	Coronal	Coronal
FA (°)	N/A	2, 5, 10, 20, 25, 30	N/A
TE (ms)	6.9	2.44	10 to 320 by 10 ms intervals
TR (ms)	4500	6.6	5000
TI (ms)	35, 75, 100, 125, 150, 250, 1000, 1500, 2000, 3000	N/A	N/A
FOV (mm^2^) (FE x PE)	250 × 250	250 × 250	250 × 250
# Slices	1	32	1
Slice thickness (mm)	6	6	6
Matrix (FE/PE/SE)	256/192/1	256/192/32	256/192/1
Number of averages	1	1	1
Acquisition time (≈min)	40	8	16

Abbreviations: IR, inversion recovery.

### 2.2 ISMRM/NIST system phantom

**FIGURE 1:**
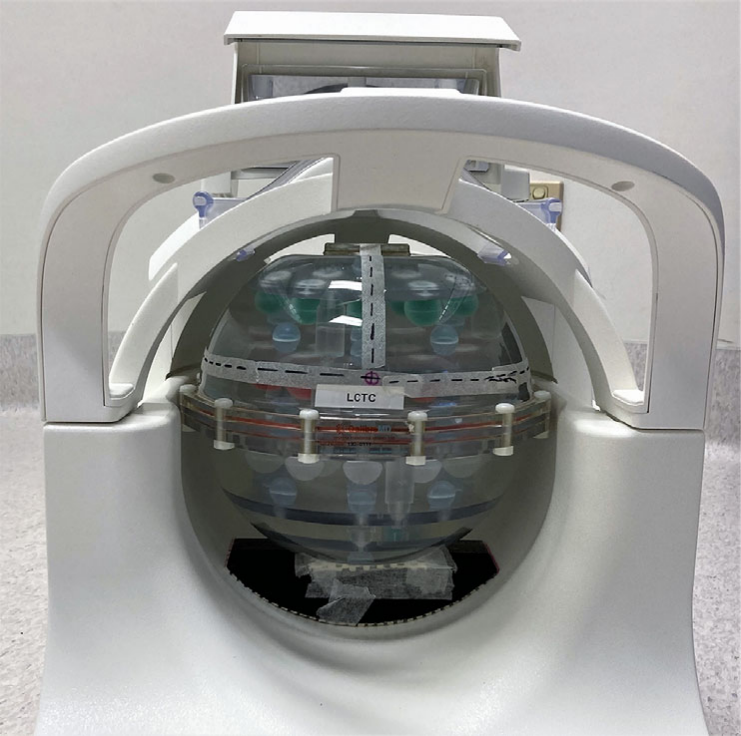
The system phantom on top of a 3D‐printed holder, fitted to the head/neck coil. 3D‐orthogonal markings were drawn to assist with external laser alignment

The phantom has a spherical geometry with a 200 mm inner diameter (ID) shell (Figure 1). The T_1_ and T_2_ sequences imaged the T_1_‐ and T_2_‐arrays embedded within the phantom, respectively. Each array contains 14 spherical (15 mm ID) vials filled with high purity water doped with either varying concentrations of NiCl_2_ (T_1_) or MnCl_2_ (T_2_). Nuclear magnetic resonance (NMR) IR methods have been used by NIST in the past to characterize these solutions under 3T and 20°C conditions (reference values provided in Table 2).^8^, ^9^ Note that vials 1 and 5 from the T_2_‐array were not included in the analysis at the recommendation of the manufacturer.

**TABLE 2:** T_1_ and T_2_ values both measured by National Institute of Standards and Technology (NIST) and experimentally derived using T_1_‐inversion recovery (IR), T_1_‐VFA or T_2_‐SE methods. Note that the NIST values were obtained from the phantom manual,^8^ and experimental values have been presented as an average of the 12 monthly measurements (with respective standard deviation [SD's]). Note the vials have been separated into full vial range (left) and human vial range (right)

Vial Number:		1	2	3	4	5	6	7	8	9	10	11	12	13	14
**T_1_ (ms) NIST**	Value	1884.0	1330.2	987.3	690.1	485.0	341.6	240.9	175.0	121.1	85.8	60.2	42.9	30.4	21.4
	SD	30.3	20.4	14.2	10.1	7.1	5.0	3.5	2.5	1.8	1.2	0.9	0.4	0.6	0.3
**T_1_‐IR (ms)**	Value	2007.2	1430.7	1032.9	655.0	489.4	348.3	246.8	170.2	117.9	86.7	64.9	46.4	36.7	36.8
	SD	77.8	63.7	12.0	13.9	5.5	1.2	1.0	4.7	3.9	4.2	1.1	0.9	3.3	6.5
**T_1_‐VFA (ms)**	Value	1894.1	1370.2	1036.0	732.7	528.3	376.6	273.5	198.1	113.9	71.1	63.3	42.2	29.7	24.4
	SD	74.7	52.4	39.8	27.1	22.7	15.8	11.9	8.4	5.4	3.9	3.4	3.7	2.1	2.4
**T_2_ (ms) NIST**	Value	*	379.5	267.3	175.1	*	88.9	63.4	44.2	29.9	19.4	14.7	10.5	7.3	5.3
	SD	*	3.6	2.5	1.7	*	0.8	0.6	0.4	0.3	0.2	0.1	0.1	0.1	0.1
**T_2_‐SE (ms)**	Value	*	364.5	269.1	183.9	*	97.1	71.9	50.2	33.4	21.2	16.0	10.0	6.3	3.5
	SD	*	7.4	5.6	3.5	*	1.8	1.3	1.0	0.8	0.6	0.4	0.4	0.4	0.4

Abbreviation: VFA, variable flip angle.

* Vials removed from analysis at the recommendation of the manufacturer.

The phantoms’ arrays covered a large range of relaxation times, including those found in the human body at 3T: T_1_ = 121 ms to 1884 ms and T_2 _= 30 ms to 79 ms.^8^, ^11^, ^18^, ^19^ The physiologic range of relaxation times was of particular interest, and thus results were separated into two categories: full vial range and, a subset, human vial range.

Temperature of the surrounding deionized water in the phantom was measured both before and after each scanning session using an NIST‐traceable thermometer (supplied with the phantom).

### 2.3 Image analysis

All image processing was completed using a consistent software platform and analysis method. ImageJ (National Institutes of Health, Maryland, USA) was initially used to manually identify the center pixel locations of each array vial on the shortest time of inversion (TI) (35 ms) image of the T_1_‐IR dataset. These locations along with all datasets were imported into an inhouse‐developed Python script. This automatically positioned a 10‐pixel (∼9.8 mm) diameter circular region of interest (ROI) to be at the center of each vial and on the central slice of the respective T_1_ and T_2_ array. The average signal for each ROI was calculated and fit to the corresponding signal equations for the respective pulse sequences (see Equations S1‐S3 and the fitting parameters provided in Table S1).

To assess the accuracy error of the measured T_1_ and T_2_ times, the %bias was calculated for each monthly acquisition and each vial using a comparison to the NIST measured reference vial value:

(1) $$\%\mathrm{bias}\;=\left(\frac{\mathrm{Measured}\;T_{1}\;-\;\mathrm{NIS}T_{\left(\mathrm{NMR}\right)}T_{1}\;}{\;\mathrm{NIS}T_{\left(\mathrm{NMR}\right)}T_{1}}\right)\;\times \;100$$

To assess the reproducibility over the range of T_1_ and T_2_ times in the phantom, a coefficient of variation (%CV) was calculated using the individual vials’ mean (μ) and standard deviation (SD) (σ), calculated over the 12 monthly repetitions:

(2) $$\%\mathrm{CV}\;=\frac{\sigma }{\mu }\;\;\times \;100$$

## 3 RESULTS

The phantoms’ T_1_ and T_2_ arrays were imaged monthly using the respective T_1_‐IR/T_1_‐VFA and T_2_‐SE imaging sequences described in Table 1. The average interval between imaging sessions was 4 weeks. The mean T_1_ and T_2_ value derived for each vial and their SDs are listed in Table 2 and were calculated using all months’ measurements. Table 2 also includes the NIST reference vial values, as reported by the manufacturer.^8^, ^9^ Figures S1–S3 display examples of model fits used to calculate these parameters.

**FIGURE 2:**
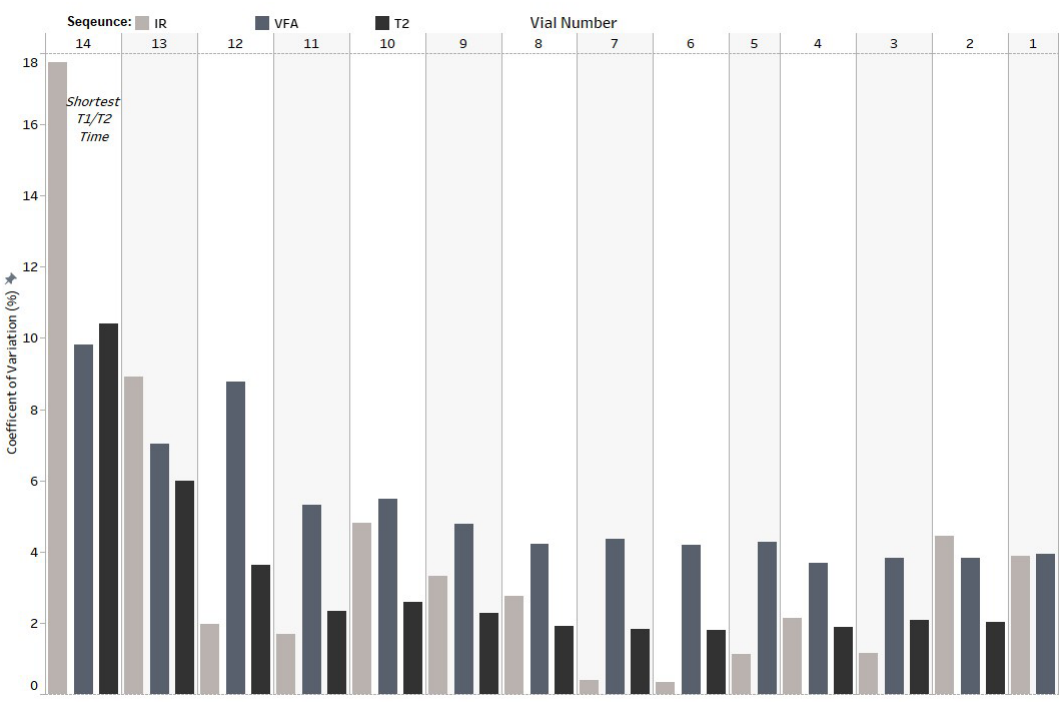
Coefficient of Variation (CV) calculated for each vial and each sequence from all monthly acquisitions. Note that vial 14 corresponded to the shortest vial reference T_1_ and T_2_ times, while vial 1 had the longest reference time

A wide range of accuracy errors in T_1_ and T_2_ measurements existed over the full vial range. Visualization of this variability can be seen in Figure S5. T_1_‐IR had the smallest bias when all vials were included (median = +3.6%), compared to T_1_‐VFA (+5.0%) and T_2_‐SE (+5.8%) sequences. In terms of reproducibility, CV's over the full vial range (Figure 2) for the same sequences respectively were 2.5%, 4.3%, and 2.2%. The largest CV's (Figure 2) and accuracy deviations were found to occur in the shortest reference time vials. For example, the T_1_‐IR bias in vials 13 and 14 was, respectively, +21% and +71%, and −14% and −35% for T_2_‐SE. Similarly, T_1_‐VFA measurements in vials 10–14 had biases in the range of −17% to +14%.

**FIGURE 3:**
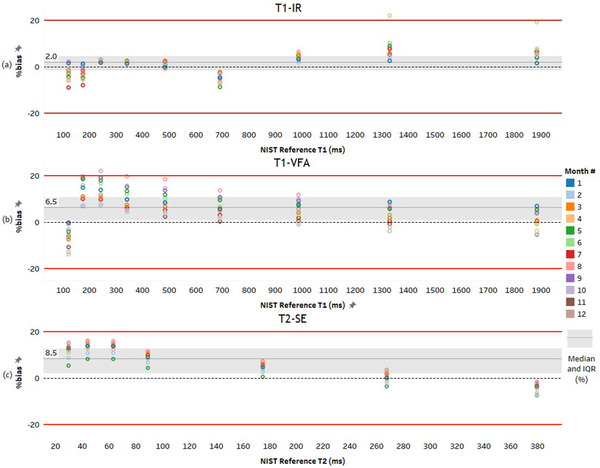
Bland–Altman plots for (a): T_1_‐IR, (b): T_1_‐VFA, and (c): T_2_‐SE. The % difference can be observed between measured and reference T_1_ and T_2_ times for vials within the human range. Median biases (and lower–upper quartiles) are displayed and include: +2.0% (−1.6–+4.5), +6.5% (+0.7–+10.8) and +8.5% (+2 ‐ +12.7) for T_1_‐IR, T_1_‐VFA and T_2_‐SE, respectively

The human vial range omitted results from the vials with the shortest reference times. Consequentially, the median bias fluctuated between approximately −20% and +20% (Figure 3), and IQR's and CV's were reduced compared to the full vial range (Table 3).

**TABLE 3:** Summary of the baseline accuracy (bias) and reproducibility (CV) measured for the 3 sequences investigated at 3T: Full phantom vial range (top) and human vial range (bottom). Values are stated as the median of all months/vials’ acquisitions and their respective inter‐quartile ranges

	(%)	T_1_‐IR	T_1_‐VFA	T_2_‐SE
Full vial range: T_1_: 1884‐21 ms T_2_: 380–5 ms	bias	+3.6 (6.9)	+5.0 (11.4)	+5.8 (14.9)
	CV	2.5 (3.0)	4.3 (1.4)	2.2 (0.9)
Human vial range: T_1_: 1884‐121 ms T_2_: 380‐30 ms	bias	+2.0 (6.11)	+6.5 (10.0)	+8.5 (10.7)
	CV	2.1 (2.2)	4.2 (0.4)	1.9 (0.2)

Abbreviations: IR, inversion recovery; CV, coefficient of variation; VFA, variable flip angle.

**FIGURE 4:**
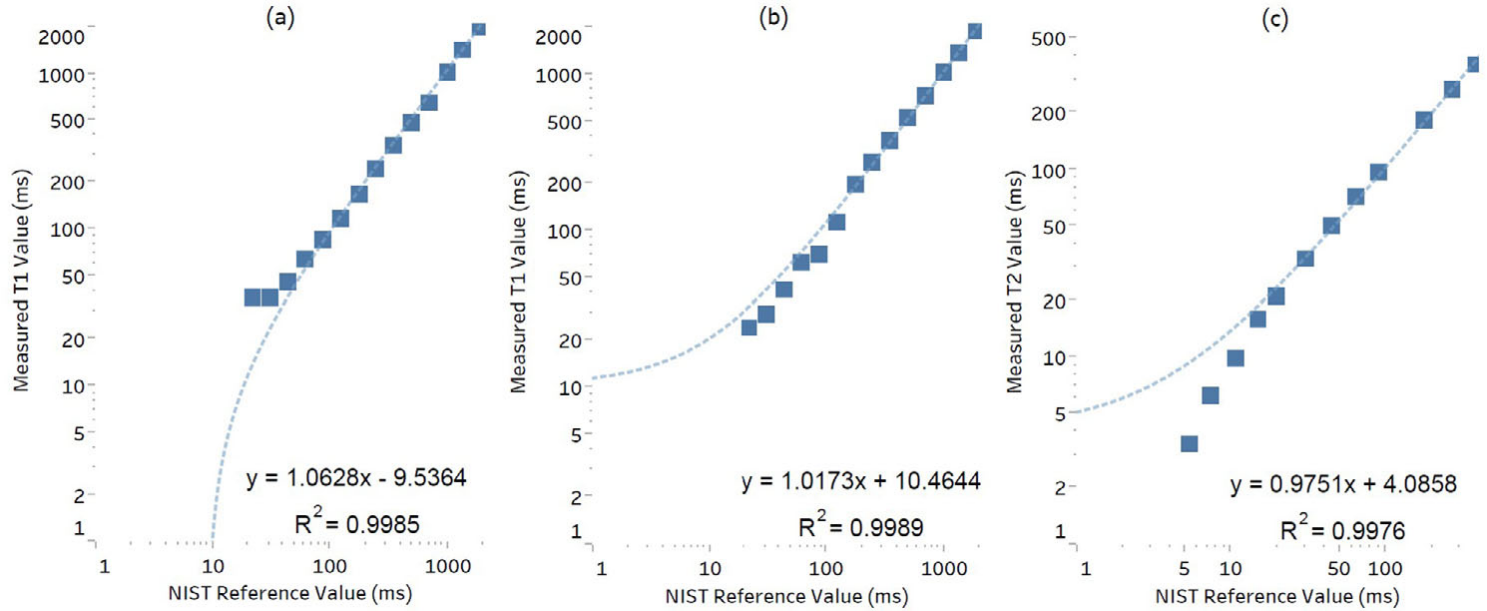
A strong linear correlation was found between (full vial range) reference and measured T_1_ and T_2_ times. This was true for (a) T_1_‐IR, (b) T_1_‐VFA and (c) for T_2_‐SE sequences. Note that all axes have employed a logarithmic scale

There was a strong linear correlation between NIST reference times and all measured relaxation times. The coefficient of determination, *R* ^2^, was calculated by plotting the reference times against those measured (Figure 4). *R* ^2^ for T_1_‐IR, T_1_‐VFA and T_2_‐SE was found to be 0.999, 0.999, and 0.998, respectively.

**FIGURE 5:**
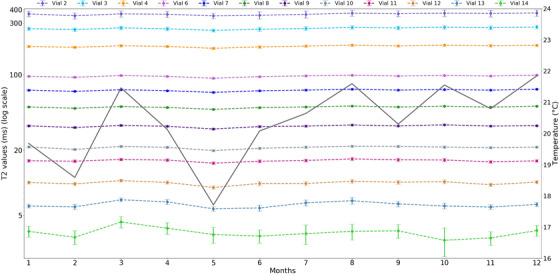
Monthly fluctuations observed in T_2_‐SE measurements, with overlaid average temperature readings. Error bars were generated from the standard deviation of each vial (calculated from the parameter fit)

Monthly changes in T_2_ measurements over the 12 months can be seen in Figure 5, along with temperature fluctuations. Each month/vial is presented with its respective errors, generated from the SD of the fit. This was calculated using the square root of the diagonals of the covariance for the parameter. Similar results for T_1_‐IR and T_1_‐VFA can be seen in Figures S8 and S9. On average, the initial and final temperature recorded each month was 20.1°C ± 1.5 °C and 20.8°C ± 1.0°C, respectively. The change in temperature over individual imaging sessions was generally less than ±0.5°C. Correlation coefficients (*ρ*) were calculated between recorded temperature and measured T_1_ for IR and VFA sequences (*ρ* = 0.003 and *ρ* = ‐0.001, respectively), and for T_2_ (*ρ* = 0.007). Similar calculations showed that there was no clear relationship between systematic variations over time with the T_1_ or T_2_ measurements (*ρ* < |0.001|).

## 4 DISCUSSION

According to the quantitative imaging biomarkers alliance (QIBA), the accuracy and precision of a quantitative parameter determine its reliability to diagnose disease or monitor a tissue response.^7^ This study was designed to assess the reliability of T_1_ and T_2_ relaxation time parameters, derived using a 3T MRI scanner.

This study expands knowledge in the field of qMRI by longitudinally monitoring samples with a wide range of T_1_ and T_2_ relaxation times at monthly measurement intervals. Accuracy and reproducibility results were comparable to previous studies completed using the same phantom type and similar sequences when including all vials.^1^, ^12^, ^14^

Note that the advice to remove specific vials from the T_2_ analysis was at the recommendation of the manufacturer. They believe there were probable mixing or labelling errors that occurred during the manufacturing of vial 5. The issues with vial 1 most likely derived from the storage of the vials’ MnCl_2_ solution prior to manufacturing the phantom: It was stored in glass, and It is suspected that the Mn within plated onto its glass storage bottle.^20^ Vial 1′s solution has a low concentration of Mn and reducing this further would result in an anomalously longer T_2_ than expected. These issues have since been resolved by the manufacturer; however, this highlights the need for monitoring qMRI systems and phantoms.

Due to limited scanner time availability, imaging could not be completed on the same day of each month. Instead, a time constraint of at least 2 weeks between imaging sessions was implemented, achieving a 4‐week average spacing. Temperature variations between 18 and 22°C had no observed effect on the measured T_1_ and T_2_ times. This was expected for the NiCl_2_ solutions in the T_1_‐array, with known minimal fluctuations within these temperature ranges.^11^ There was a 1.6%/°C linear dependence expected for the MnCl_2_ T_2_‐array solutions.^11^ However, due to the small temperature fluctuations recorded in this study (averaged within 1°C of the NIST reference conditions), no significant relationship was observed (*ρ* = 0.007).

Reproducibility was improved for T_1_ and T_2_ measurements in the human vial range of the phantom compared to the full range. The CV of T_1_ and T_2_ in this range for all sequences tested was less than 5%. Further, Bland–Altman plots in Figure 3 showed the bias of these parameters ranged between approximately −20% and +20%, with an average parameter over‐estimation. The average of the biases (+2.0%, +6.5% and +8.5% for T_1_‐IR, T_1_‐VFA and T_2_‐SE, respectively) were far less in magnitude (<20%) than those likely to cause erroneous outcomes if used in applications like tissue discrimination (e.g., benign vs. malignant).^2^, ^19^

T_1_‐IR, a gold standard T_1_‐mapping method, had a greater accuracy and reproducibility compared to T_1_‐VFA, in agreement with the literature.^1^, ^19^ This was expected as VFA methods are known to overestimate T_1_, along with have increased sensitivity to B1‐inhomogenieity effects compared to IR and often require a correction technique.^1^ Clinically, T_1_‐VFA with 2–3 flip angles is preferred over IR methods due to shorter acquisition times.^7^, ^10^ This study aimed to follow QIBA guidelines by utilizing a common imaging protocol that was open source and could allow for prospective multi‐site investigations.^7^, ^17^ Note that no B1‐corrections were implemented in this study as there is currently no commonly used correction technique available.^21^ A future study would utilize department‐specific patient imaging protocols for T_1_‐mapping and compare scanner baseline %bias and reproducibility.

There was a strong linear correlation between the reference and measured vial relaxation times (*R* ^2 ^> 0.997). It can be seen in Figure 4 and Figure S5 that the largest deviations in %bias and reproducibility occurred for vials with the smallest relaxation times. This can be partially explained by the acquisition parameters utilized. For example, in the T_1_‐array, vials 13 and 14 had reference times of approximately 30 ms and 21 ms, respectively; shorter than that of the first TI (35 ms) used in the T_1_‐IR pulse sequence. Similarly, for the T_2_‐array, vials 13 and 14 had reference times of 7 ms and 5 ms; less than the shortest TE (10 ms) used in the T_2_‐SE sequence. Detecting shorter T_1_ and T_2_ times is often a challenge to scanner's gradient hardware and available sequence acquisition parameters.^12^ However, these sequences were utilized as they are commonly available and designed to capture the wide range of relaxation times in the phantom.

For the T_2_‐array, signals for shorter T_2_ vials often approached the noise floor (Figure S3). Also, the mono‐exponential fitting applied to the T_2_‐SE signal, replicating methods used in the majority of clinical and preclinical studies, is known to be susceptible to inaccuracies generated by B1‐inhomogeneities.^13^, ^19^, ^22^ This can lead to imperfect refocusing flip angles, especially for the first echo (Figure S4) and can contribute noise.^5^, ^22^, ^23^ For these reasons, the first TE and signal were discarded from the fit, and a noise factor was introduced in the model fitting procedure (see Equation S3).

During post‐processing of the T_1_‐VFA magnitude images, the average signal from ROI's in vials with shorter reference times (9‐14) was observed to have signal saturation. This was especially the case for larger flip angles (20°–30°). Thus, only magnitude images for FA's 2°, 5°, and 10° were used to calculate T_1_ for the saturated vials, similar to Keenan et al. (Figures S2, S6, and S7).^14^

Figures S8 and S9 and Figure 5 show no trends in variability for T_1_ or T_2_ accuracy measurements over the course of the 12‐month study. According to the literature, major system upgrades can cause large changes to occur in T_1_ measurements.^14^, ^24^, ^25^ During this study, two hardware replacements of the Transmit‐Box (containing RF transmitters) occurred between months 7 and 8 and also months 10 and 11. Although no correlation between system upgrades and relaxation times were found, in T_1_‐VFA measurements, the percentage pixels with signal saturation reduced in months 8 (−3.4%) and 11 (−2.3%) (Figure S6). However, this was not significantly different when compared to other months, and hence the cause of the reduction was not determined.

With the high repeatability of the accuracy measurements observed, similar to Ihalainen et al., it is predicted that future measurements using this scanner would yield similar results.^15^ Consequentially, QA frequency recommendations to the department involved conducting testing annually and surrounding the time of any major scanner upgrades. A future investigation would conduct similar measurements at daily intervals over 1 month to determine if any fluctuations occur in between the monthly measurements. These longitudinal and frequent assessments of qMRI scanner technical performance fluctuations are especially important in the case of treatment response monitoring.^7^

## 5 CONCLUSION

In conclusion, our study found high accuracy and long‐term reproducibility in physiologically relevant T_1_ and T_2_ times on a radiotherapy dedicated 3T MRI scanner. Baseline bias (and CV [%]) for T_1_‐IR, T_1_‐VFA and T_2_‐SE sequences were +2.0% (2.1), +6.5% (4.2), and +8.5% (1.9) respectively. Shorter sample relaxation time vials had increased measurement instability; however, no systematic variations in accuracy over time were observed. For this scanner, it was recommended that only annual qMRI QA measurements need be taken combined with before and after any major scanner upgrades following baseline establishment. Further investigations are required to determine deviations in T_1_ and T_2_ when using department‐specific sequences and to find the cause of the signal saturation fluctuations in T_1_‐VFA acquisitions for shorter reference time vials.

## DISCLAIMER

Certain commercial equipment, instruments, or materials are identified in this paper in order to specify the experimental procedure adequately. Such identification is not intended to imply recommendation or endorsement by NIST, nor is it intended to imply that the materials or equipment identified are necessarily the best available for the purpose.

## CONFLICT OF INTEREST

The authors declare that there is no conflict of interest that could be perceived as prejudicing the impartiality of the research reported.

## AUTHOR CONTRIBUTIONS

Madeline Carr performed the measurements, analyzed the data, and wrote the manuscript with support from all the authors. Kathryn Keenan, Robba Rai, Peter Metcalfe, Amy Walker, and Lois Holloway were involved in experiment planning and supervising the work.

## Supporting information

Supporting information
