# Supplementary material for: Determining the longitudinal accuracy and reproducibility of T1 and T2 in a 3T MRI scanner
Source: J Appl Clin Med Phys. 2021 Sep 25;22(11):143–50. doi: 10.1002/acm2.13432 (PMC8598150; doi:10.1002/acm2.13432)
Supplement: Supplementary file 1 — Supporting information [file ACM2-22-143-s001.docx]

**Supplementary Material**

**Method:**

For each monthly dataset, the signal average (S) was derived from each of the vials’ ROI. This was then fitted to signal Equation (1), (2) or (3) depending on the sequence: T_1_-IR, T_1_-VFA or T_2_-SE, respectively. Note for T_1_-VFA signal fitting, vials 1-8 were fit with all six flip angles (FAs) in the image sequence. Vials 9-14 however, due to the large amount of signal saturation for FAs 20-30 ^o^, were only fit with FAs: 2 ^o^, 5 ^o^ and 10^o^. This was a similar filtering method to that employed by Keenan et al [1].

The resulting fitted signal from month 1 to signal Eqs. (1-3) below are shown in Supplementary Figures S1-S3 (similar fits were observed for all months). Results of these fits over the full vial range can be seen in Supplementary Figure S5. Further, the extent of saturated pixels in the T_1_-VFA data is outlined in Supplementary Figures S6 and resulting fitted T_1_ times for these vials (without using the filtering process: i.e., using 6 FAs for all vials) is displayed in Supplementary Figures S7. Lastly, initial parameters and bounds for each of the fits have been outlined in Supplementary Table S1.

T_1_-IR: $S\left( TI \right)= \left| M_{0}(1+(invF-1)e^{\frac{-TR}{T_{1}}}-invF\cdot e^{\frac{-TI}{T_{1}}}) \right|+n$ [1]

T_1_-VFA: $S\left( \alpha\right)=\frac{\sin\left( \alpha\right)\cdot M_{0}[1-e^{\frac{-TR}{T_{1}}}]}{1-cos(\alpha)\cdot e^{\frac{-TR}{T1}}}$ [2]

T_2_-ME: $S\left( TE \right)={M_{0}e}^{\frac{-TE}{T2}}+n$ [3]

Here, $\alpha$ was the nominal flip angle (FA) used in the VFA acquisition, $TR$ was the repetition time, $TI$ was the time of the inversion pulse and $TE$ was the echo time. These parameters were read from the DICOM header in the imported images using Python. Also, $invF=(1-\cos\left( \emptyset\right))$is the inversion factor where $\emptyset$ is the angle of inversion and $a$,$M_{0}$ (equilibrium magnetization) and $n$(noise factor) were unknown parameters estimated using a nonlinear least-squares fitting method [1, 2, 3, 4].

This process included a mono-exponential fit (3-parameters) for the T_2_-SE signal whereby the first echo was omitted due to expected and observed inconsistent phase coherences [5, 6]. Observations of error introduced by the 1^st^ echo can be seen in Supplementary Figure S4. For T_1_-IR (4-parameter model) and T_1_-VFA (2-parameter), similar initial parameters and bounds were implemented to those described in Bane et. al. [4].

**Supplementary Table:**

| **Sequence** | **Parameter** | **Initial Value** | **Min** | **Max** |
| --- | --- | --- | --- | --- |
| **T_1_-IR** | $M_{0}$ | max ($S\left( TI \right)$) | 0 | - |
|  | $invF$ | 2 | 1 | 2 |
|  | $n$ | 0 | 0 | - |
|  | $T1$ (ms) | TI_null_/ln(2) | 0 | 5000 |
| **T_1_-VFA** | $M_{0}$ | max ($S\left( \alpha\right)$) | 0 | - |
|  | $T1$ (ms) | 800 | 10 | 3000 |
| **T_2_-SE** | $M_{0}$ | max($S\left( TE \right)$) | 0 | - |
|  | $n$ | 0 | - | - |
|  | $T2$ (ms) | median($S\left( TE \right)$) | 0 | 2000 |

Supplementary Table S1: Initial values and bounds for parameters implemented into the 3 sequence signal fits. Note that TI_null_ was estimated as the TI of which had the minimum signal ($S\left( TI \right)$) value registered to the T_1_-IR data.

**Supplementary Figures:**

Supplementary Fig S1: T_1_-IR fit produced for vials 1-14 for month 1. Values are given as T_1_ ± SD (of the fit). No signals approached saturation points (magnitude = 4095).


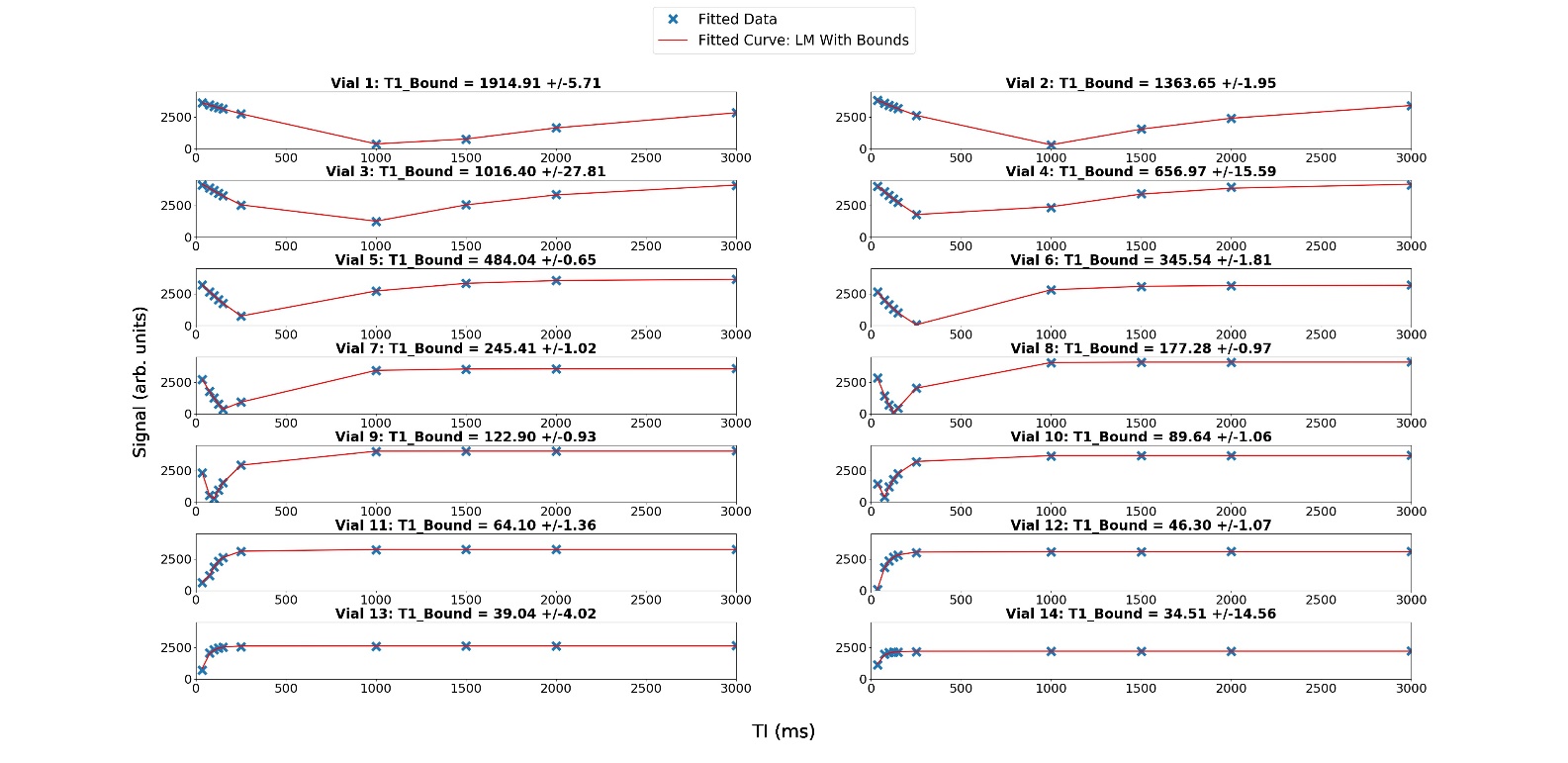


Supplementary Fig S2: T_1_-VFA fit produced for vials 1-14 for month 1. Values are given as T_1_ ± SD (of the fit). Notice the green data points approaching saturation (magnitude = 4095) at shorter reference T_1_ vials (9-14) and for larger flip angles. These were removed from the fit as seen.

. Note the approach to the saturated pixel values for lower vials (10-14).


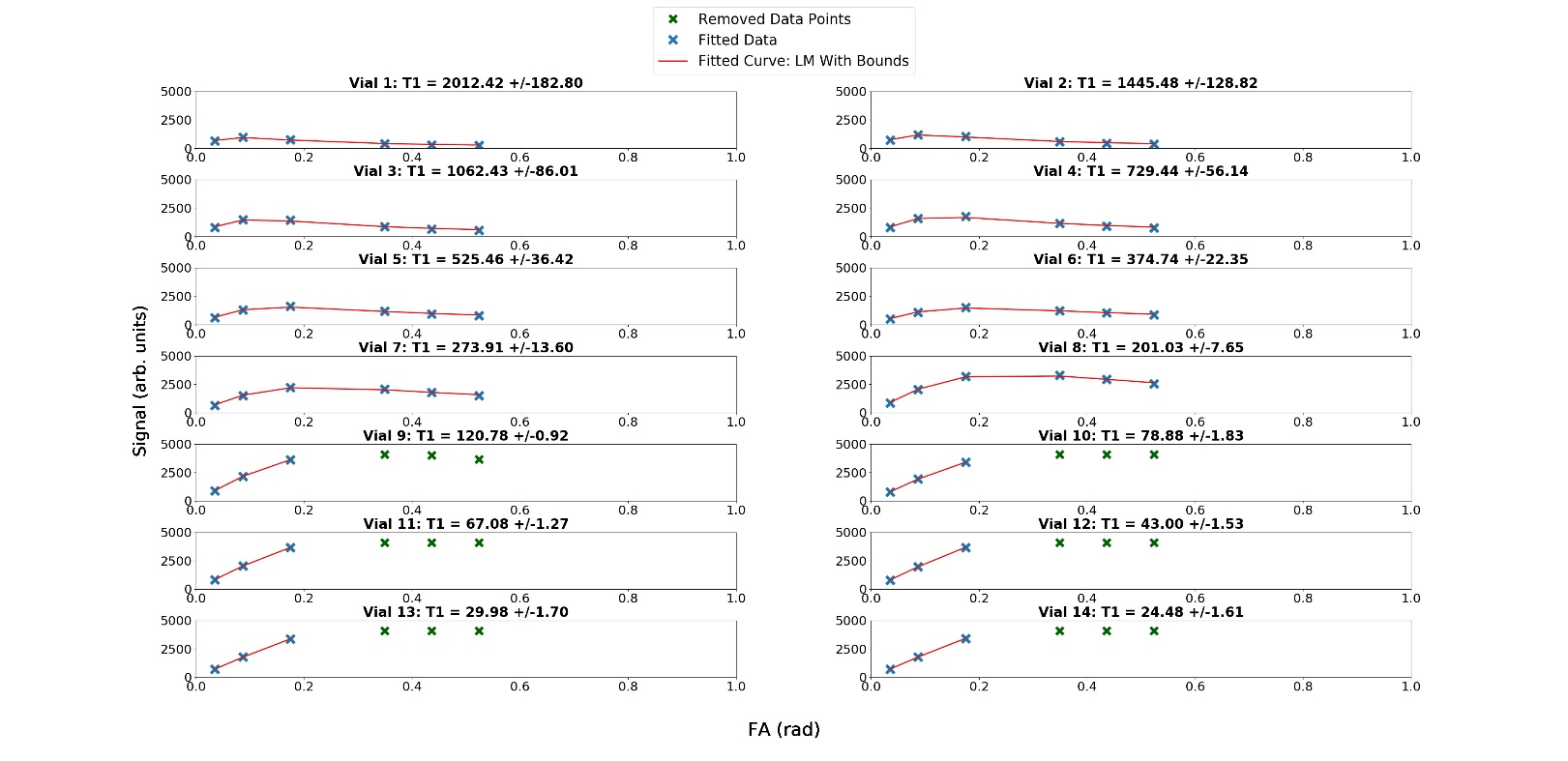


Supplementary Fig S4: Original T_2_-SE fit produced for vials 1-14 for month 1 whilst preserving the 1^st^ echo. Values are given as T_2_ ± SD (of the fit). Note the difficulty in producing the mono-exponential fit; large SD error were produced along with illogical overestimations of T_2_, especially for higher reference time vials (1-4). Note vials 1 and 5 were removed from the main analysis at the recommendation of the manufacturer.

**NEED to replace this graph keeping first echo**


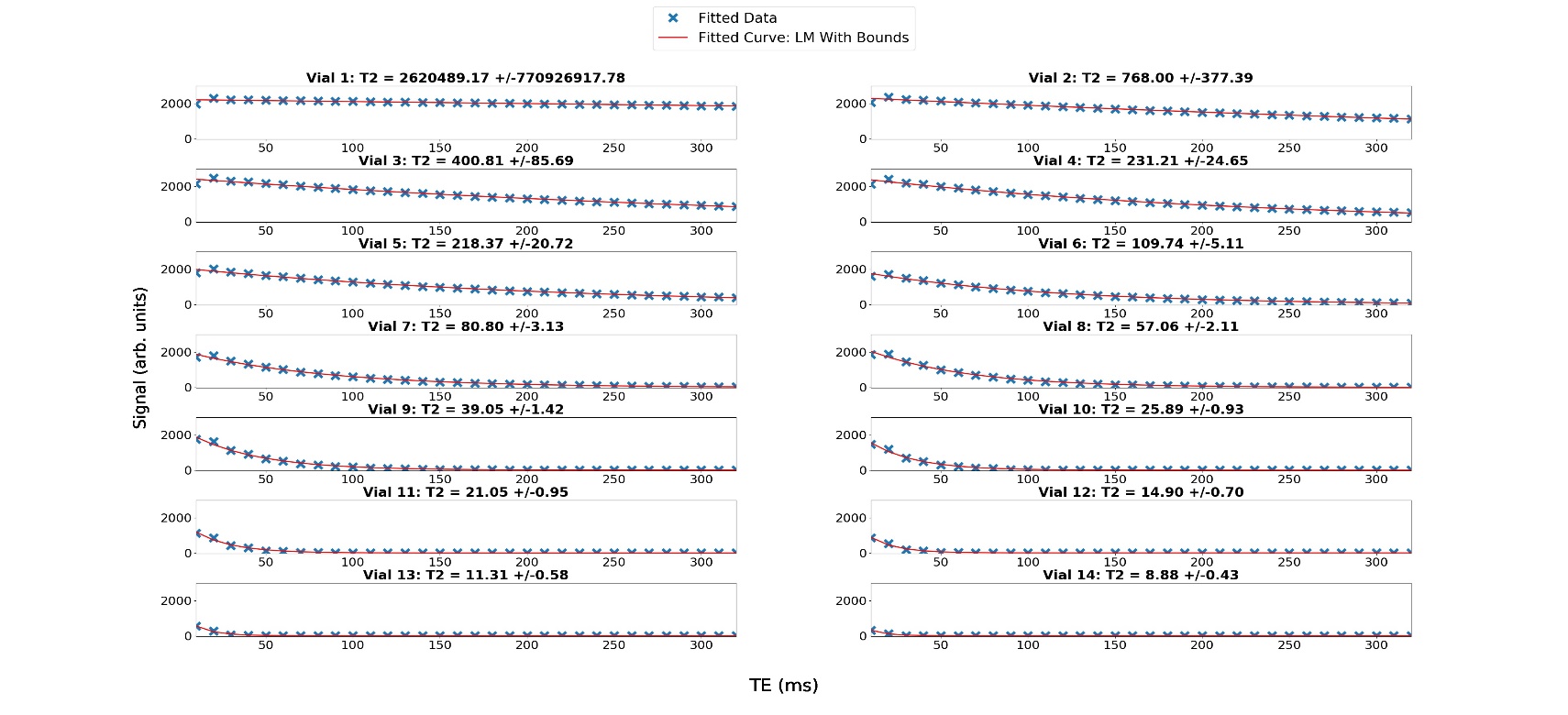

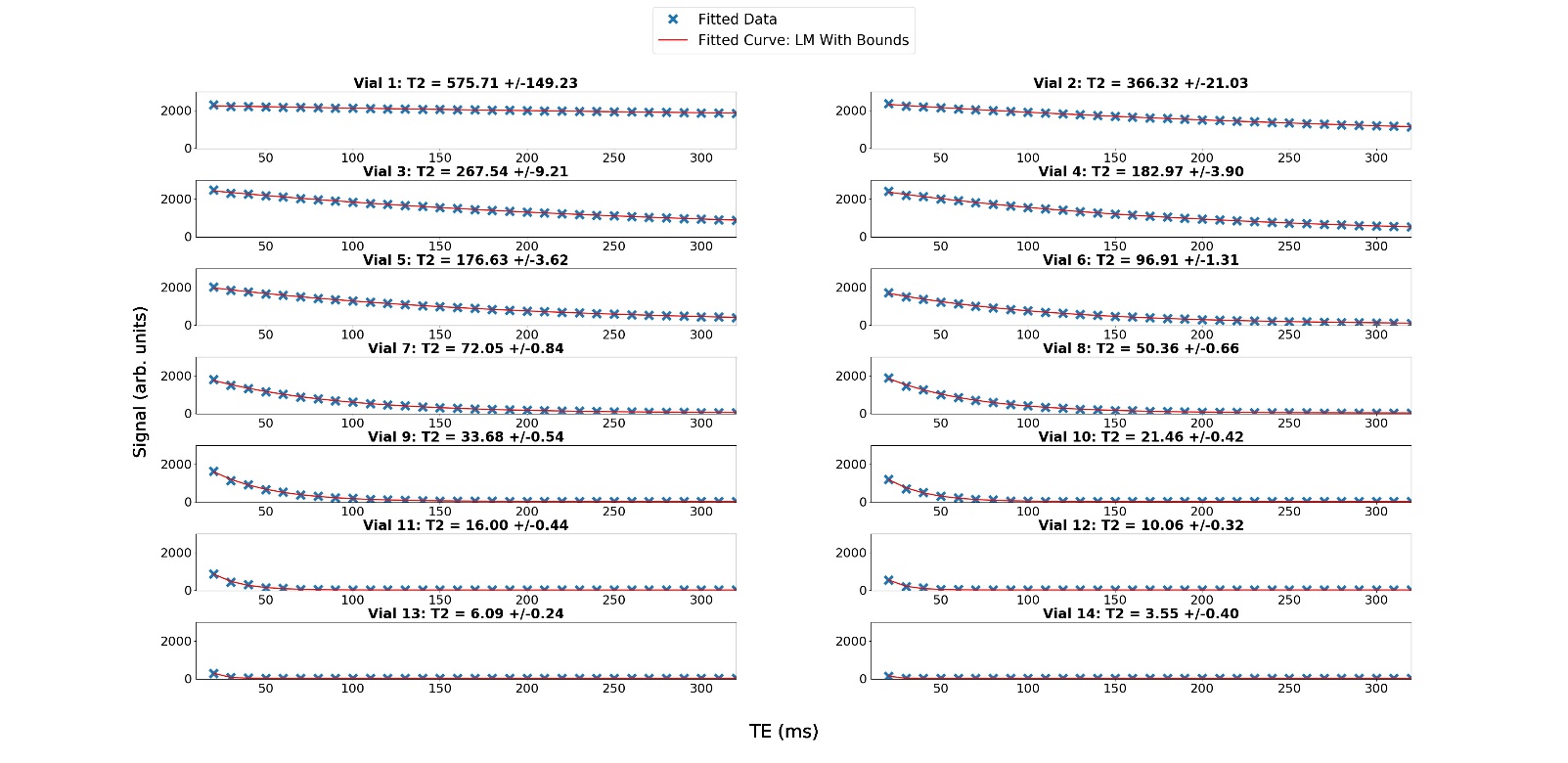


Supplementary Fig S3: T_2_-SE fit produced for vials 1-14 for month 1. Values are given as T_2_ ± SD (of the fit). Here, the 1^st^ echo has been removed and signals were approaching the noise floor, especially for shorter reference T_2_ vials. Note vials 1 and 5 were removed from the main analysis at the recommendation of the manufacturer.

Supplementary Fig S5: Bland-Altman plots for (a) T_1_-IR, (b) T_1_-VFA and (c) T_2_-SE (bottom), show the difference (%) between measured and reference T_1_ and T_2_ times for vials in the full vial range. Median bias (and lower – upper quartiles) are displayed and include: +3.6 % (+0.8 – +7.7), +5.0% (-1.46 – +9.96) and +5.8% (-3.8 – +11.1) for T_1_-IR, T_1_-VFA and T_2_-SE respectively.


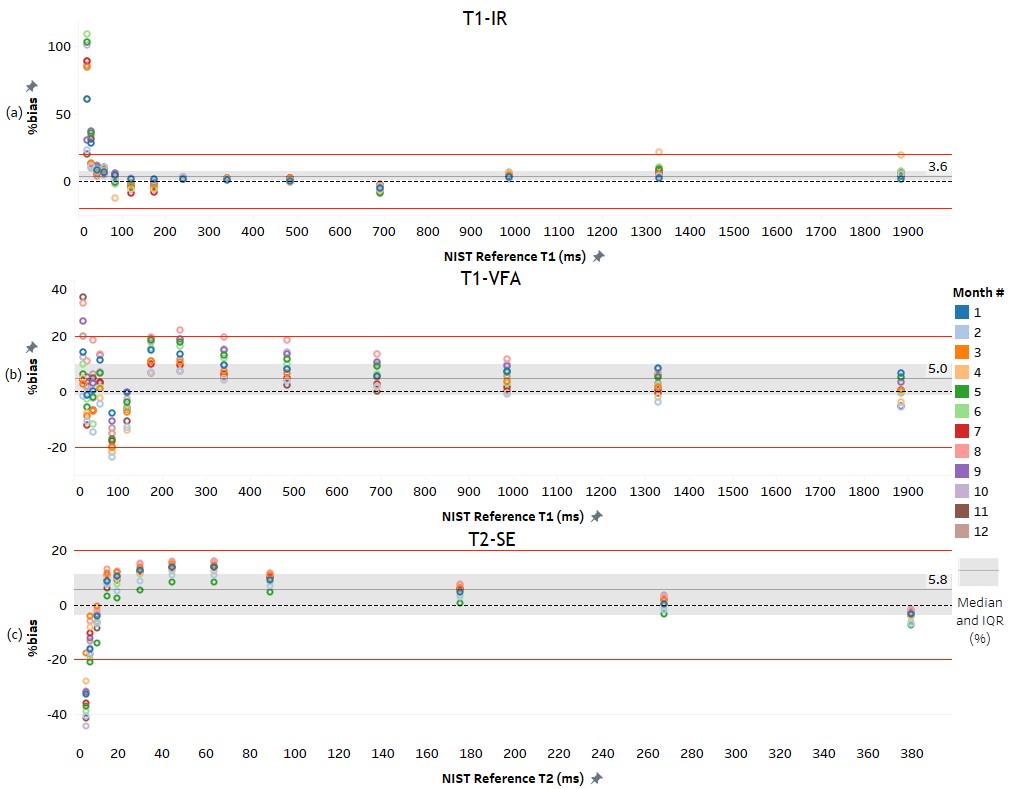

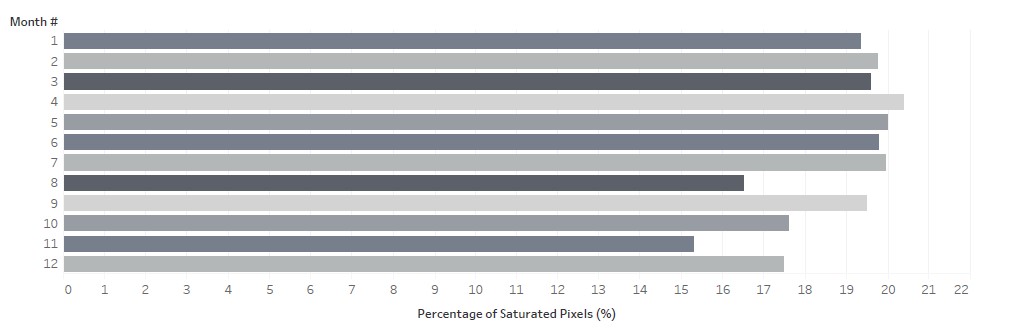


Supplementary Fig S6: Variability in the percentage of saturated pixels in T_1_-VFA data (out of a potential 5796 pixels per month: 69 pixels x 14 vials x 6 FA’s) observed over the 12-month study in all vials. No saturation occurred in vials 1-8, and in general, saturation was only seen for FA’s 20, 25 and 30 degrees. Note that the TX-box upgrade occurred between months 7 and 8, and again between months 10 and 11. We note the decrease in percentage of saturated pixels in months’ 8 and 11.

Supplementary Fig S7: Non-filtered Bland-Altman plots for T_1_-VFA highlights the difference (%) between measured and reference T_1_ times for vials in the full range. Notice the large variability in %bias for shorter T_1_-times. Median biases (and lower – upper quartiles) are displayed and include: +11.7 % (+5.5 – +89.9).


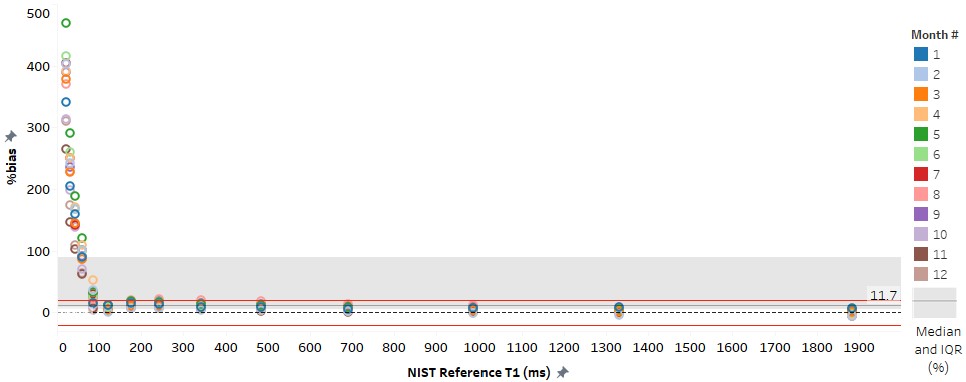

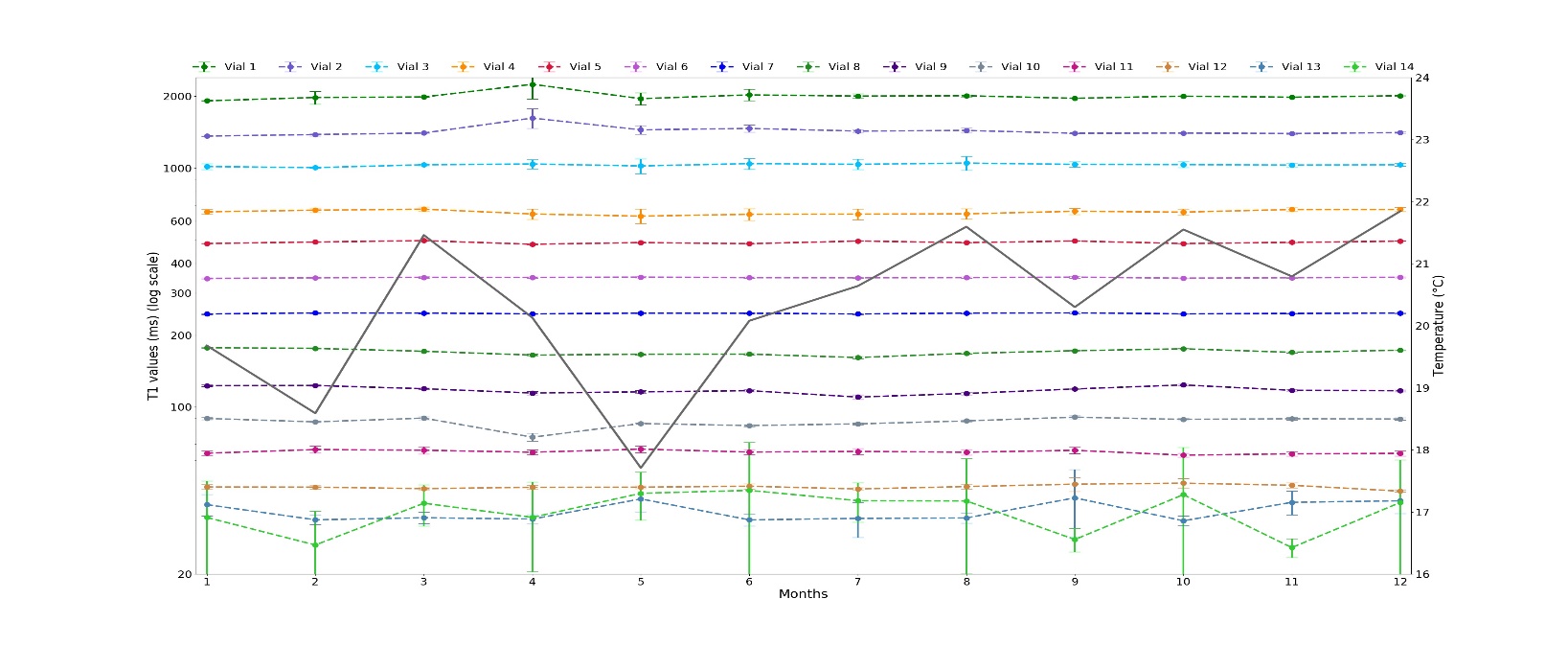


Supplementary Fig S8: Monthly fluctuations observed in T_1_-IR measurements with overlaid average temperature readings. Error bars were generated from the standard deviation of each vial (calculated from the parameter fit).

Supplementary Fig S9: Monthly fluctuations observed in T_1_-VFA measurements with overlaid average temperature readings. Error bars were generated from the standard deviation of each vial (calculated from the parameter fit).


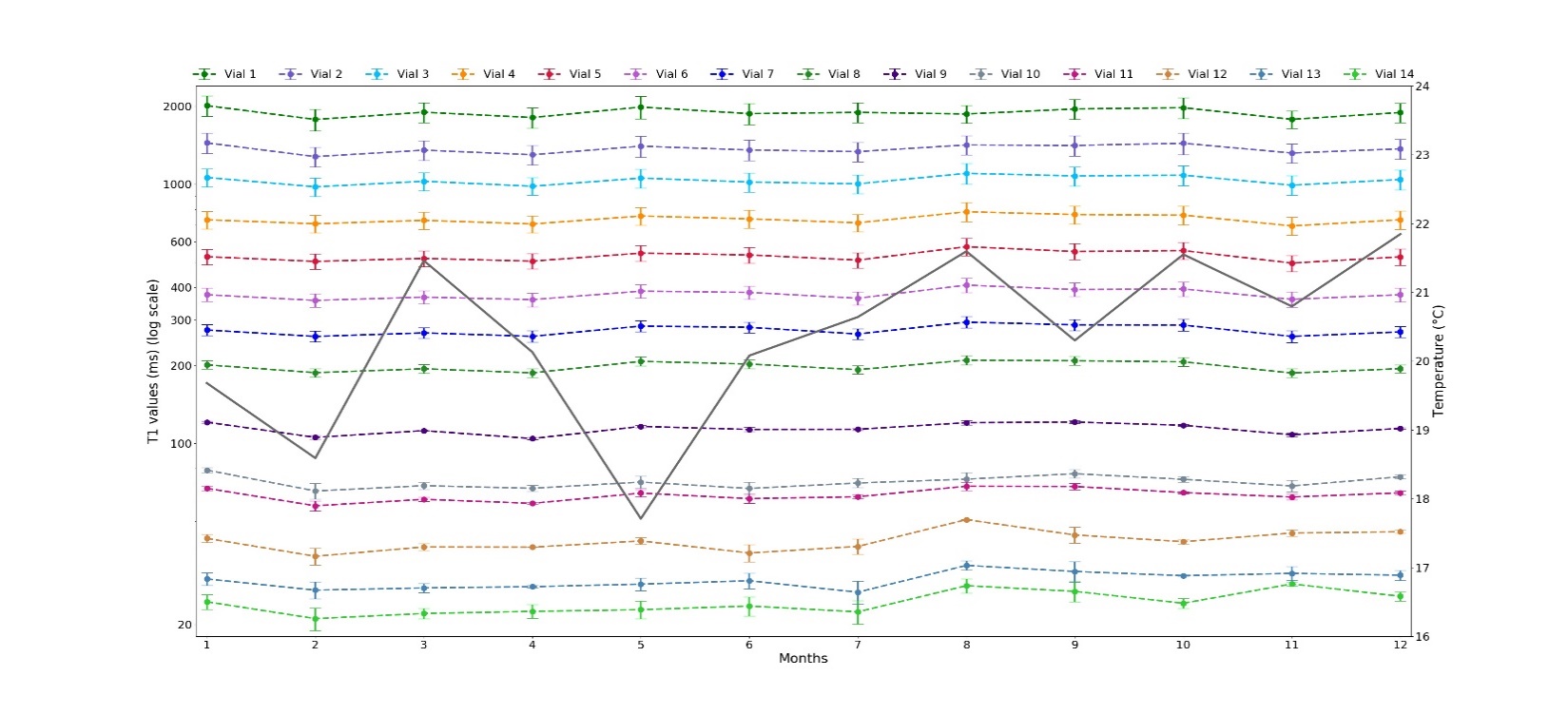


References:

| [1] | Keenan KE, Gimbutas Z, Dienstfrey A, Stupic KF. Assessing effects of scanner upgrades for clinical studies. *J Magn Reson Imaging.* 2019;50: 1948-1954. [doi:10.1002/jmri.26785](https://doi.org/10.1002/jmri.26785) |
| --- | --- |
| [2] | Tofts P, Steens S*. Quantitative MRI of the brain: Measuring changes caused by disease*. John Wiley & Sons Ltd*.* 2003. |
| [3] | Jiang Y, Ma D, Keenan KE, Stupic KF, Gulani V, Griswold MA. Repeatability of magnetic resonance fingerprinting T1 and T2 estimates assessed using the ISMRM/NIST MRI system phantom. *Magn Reson Med.* 2017 Oct;78(4):1452-1457. [doi:10.1002/mrm.26509](https://doi.org/10.1002/mrm.26509). |
| [4] | Bane O, Hectors SJ, Wagner M, Arlinghaus LL, Aryal MP, Cao Y et al.. Accuracy, repeatability, and interplatform reproducibility of T1 quantification methods used for DCE-MRI: Results from a multicenter phantom study*. Magn Reson Med*. 2018 May;79(5):2564-2575. [doi:10.1002/mrm.26903](https://doi.org/10.1002/mrm.26903). |
| [5] | Milford D, Rosbach N, Bendszus M, Heiland S. Mono-Exponential Fitting in T2-Relaxometry: Relevance of Offset and First Echo. *PLoS One*. 2015;10(12):e0145255. doi:10.1371/journal.pone.0145255. |
| [6] | McCann A, Wilson P, McGrath C. Initial Experiences with the ISMRM / NIST Quantitative MRI ‘System Standard’ Phantom. IPEM- Quantitative MRI: Clinical Applications and Quality Assurance, Belfast, 2018. |
|  |  |
|  |  |
|  |  |
|  |  |
